# Genomic epidemiology and population structure of *Neisseria gonorrhoeae* from remote highly endemic Western Australian populations

**DOI:** 10.1186/s12864-018-4557-5

**Published:** 2018-02-27

**Authors:** Barakat A. Al Suwayyid, Geoffrey W. Coombs, David J. Speers, Julie Pearson, Michael J. Wise, Charlene M. Kahler

**Affiliations:** 1 The Marshall Centre for Infectious Diseases Research and Training, The University of Western Australia, Crawley, Australia; 2 School of Biomedical Sciences, Faculty of Health and Medical Sciences, The University of Western Australia, Crawley, Australia; 3 Ministry of Education, Riyadh, Saudi Arabia; 4 School of Veterinary and Life Sciences, Murdoch University, Murdoch, Australia; 5 Department of Microbiology, Pathwest Laboratory Medicine WA, Fiona Stanley Hospital, Murdoch, Australia; 6 Department of Microbiology, Pathwest Laboratory Medicine WA, Queen Elizabeth II Medical Centre, Nedlands, Australia; 7 School of Medicine and Pharmacology, University of Western Australia, Crawley, Australia; 8 Computer Science and Software Engineering, The University of Western Australia, Crawley, Australia

**Keywords:** *Neisseria gonorrhoeae*, Gonorrhoea, Whole genome sequencing, Population structure, Western Australia, Australia

## Abstract

**Background:**

*Neisseria gonorrhoeae* causes gonorrhoea, the second most commonly notified sexually transmitted infection in Australia. One of the highest notification rates of gonorrhoea is found in the remote regions of Western Australia (WA). Unlike isolates from the major Australian population centres, the remote community isolates have low rates of antimicrobial resistance (AMR).

Population structure and whole-genome comparison of 59 isolates from the Western Australian *N. gonorrhoeae* collection were used to investigate relatedness of isolates cultured in the metropolitan and remote areas. Core genome phylogeny, multilocus sequencing typing (MLST), *N. gonorrhoeae multi-antigen sequence typing (*NG-MAST) and *N. gonorrhoeae* sequence typing for antimicrobial resistance (NG-STAR) in addition to hierarchical clustering of sequences were used to characterize the isolates.

**Results:**

Population structure analysis of the 59 isolates together with 72 isolates from an international collection, revealed six population groups suggesting that *N. gonorrhoeae* is a weakly clonal species. Two distinct population groups, Aus1 and Aus2, represented 63% of WA isolates and were mostly composed of the remote community isolates that carried no chromosomal AMR genotypes. In contrast, the Western Australian metropolitan isolates were frequently multi-drug resistant and belonged to population groups found in the international database, suggesting international transmission of the isolates.

**Conclusions:**

Our study suggests that the population structure of *N. gonorrhoeae* is distinct between the communities in remote and metropolitan WA. Given the high rate of AMR in metropolitan regions, ongoing surveillance is essential to ensure the enduring efficacy of the empiric gonorrhoea treatment in remote WA.

**Electronic supplementary material:**

The online version of this article (10.1186/s12864-018-4557-5) contains supplementary material, which is available to authorized users.

## Background

*Neisseria gonorrhoeae* (gonococcus), the causative agent of gonorrhoea, is one of the most common causes of bacterial-associated sexually transmitted infection (STI). Estimated to cause 78 million new infections worldwide, 35 million cases of *N. gonorrhoeae* occur annually in the Western Pacific Region which includes Australia [1]. Australian states and territories are divided into remote and metropolitan areas, based on the distance to the closest service centers and the population density. Communities living in remote regions of the NT and WA have the highest notification rate of gonorrhoea in Australia [2, 3]. In 2015, gonorrhoea notifications were reported to be 959 per 100,000 population of people living in the remote WA Kimberley region, compared to 85 per 100,000 population for Western Australia [4].

Gonococcal vaccines are currently not available and antimicrobial therapy is the only effective option for treating gonorrhoea. However, the World Health Organization (WHO) Global Gonococcal Antimicrobial Surveillance Programme (GASP) has reported the efficacy of many antibiotic treatments such as penicillin, tetracycline, and ciprofloxacin is lower than 95% indicating these antibiotics can no longer be used for empirical treatment in almost all GASP countries [5]. This has resulted in a shift towards dual antimicrobial therapy, mainly ceftriaxone and azithromycin. Among WHO GASP countries, isolates with decreased susceptibility or resistance to the extended-spectrum cephalosporins (ESCs) and isolates with resistance to azithromycin have been reported in 51% and 75% of these countries respectively [6]. To understand the epidemiology underlying the appearance and spread of antimicrobial resistance (AMR) in gonococci, several molecular approaches such as whole genome sequencing (WGS), multilocus sequence typing (MLST), *N. gonorrhoeae* multi-antigen sequence typing (NG-MAST) and most recently *N. gonorrhoeae* sequence typing for antimicrobial resistance (NG-STAR) have been developed [7–9]. Resistance to ESCs has spread primarily through clonal expansion and is highly correlated with the presence of the *penA* mosaic allele in NG-MAST sequence type (ST)1407, MLST STs 1901 and 7363 and NG-STAR ST90 clusters [8, 10, 11]. Similarly, emergent quinolone resistant strains have spread clonally in the NG-MAST ST225 lineage which is associated with *gyrA* and *parC* chromosomal mutations [11, 12]. Azithromycin resistance, which initially appeared sporadically, has rapidly spread through local sexual networks in geographically distinct regions [13]. Multi-drug resistance (MDR) has been increasing clonally through a limited number of STs and phylogenetic clusters in the last 4 years with resistance to cephalosporins and azithromycin emerging a limited number of times [12, 14, 15].

The prevalence of AMR amongst gonococcal isolates in metropolitan areas of WA is similar to global levels, with 20% of isolates being penicillin and ciprofloxacin resistant; while ESC decreased susceptibility and azithromycin resistant isolates are sporadically reported [16]. In contrast, the prevalence of AMR amongst isolates from the remote areas of WA is lower at 2–3% for penicillin and ciprofloxacin resistance while no resistance to ESCs or azithromycin has been detected [16, 17]. The lack of AMR in isolates from remote regions of Australia may be explained by the limited international contacts and by recent studies utilizing NG-MAST and MLST that have revealed gonococcal isolates in the remote areas are genetically distinct from metropolitan isolates which may be due to the different risk groups in each area: men who have sex with men (MSM) networks from the metropolitan areas versus the heterosexual indigenous communities living in remote areas [18, 19].

Our study aims to elucidate the genomic epidemiology and population structure of *N. gonorrhoeae* in WA using isolates collected from 2011 to 2013. Moreover, the study aims to determine the association between the *N. gonorrhoeae* types found in WA according to the molecular typing schemes and their antimicrobial susceptibility.

## Methods

### Bacterial isolates

In WA, gonorrhoea is a Department of Health notifiable infectious disease and all isolates are referred to the Western Australian Gonococcal Surveillance Programme (WAGSP) laboratory for antimicrobial susceptibility testing. Antimicrobial resistance profiles of all isolates to penicillin, spectinomycin, azithromycin, ciprofloxacin, ceftriaxone and high-level resistance to tetracycline (tetracycline HLR) were assessed by the agar dilution method and interpreted using the Calibrated Dichotomous Sensitivity guidelines [20]. Decreased susceptibility to ceftriaxone (0.06–0.126 mg/L) is confirmed by the E-test (bioMérieux, France). Penicillinase production is detected using the nitrocefin test (Oxoid, Australia). Anatomical isolation site, geographical location and postcode are available from the Communicable Disease Control Directorate.

Fifty nine *N. gonorrhoeae* isolates from patients living in the remote (*n* = 33) and metropolitan population centers (*n* = 26) of WA from 2011 and 2013 were obtained from the WAGSP laboratory [16]. Isolates were stored in GC broth with 20% glycerol at − 80 °C and were passaged fewer than five times and were cultured under aerobic conditions with 5% CO_2_ at 37 °C on GC agar (Oxoid, Australia) supplemented with 0.4% glucose, 0.01% glutamine, 0.2 mg/L of cocarboxylase and 5 mg/L of iron (III) nitrate.

### DNA extraction, genome sequencing and assembly

Genomic DNA extraction was performed using the DNeasy Blood and Tissue Kit (Qiagen, Germany) and the extract was stored as per the manufacturer’s instructions for DNA extraction from Gram negative bacteria. Genome sequencing of the 59 isolates was performed using the Illumina MiSeq platform (Illumina, USA) with 2 × 300 base pair read lengths. The targeted sequencing depth was 120 with a minimum Phred quality score of 30. Reads were de novo assembled using SPAdes genome assembler version 9.0 [21]. The quality of the assembled genomes was assessed using the Quast genome assembly evaluation tool [22]. Sequencing and assembly quality statistics of the 59 WA *N. gonorrhoeae* isolates are shown in Additional file 1. Bacterial Isolates Genome Sequence database (BIGSdb) genomics platform tools – hosted on www.pubmlst.org/neisseria – were used for annotation and genome wide analysis of the assembled isolates [23]. The core genome consisted of 1427 genes that were present in all of the 59 *N. gonorrhoeae* isolates included in this analysis. The core genome was determined using the genome comparator tool at BIGSdb using the “all loci scheme” with 100% core threshold and excluding incomplete loci. Once the analysis was completed a list of complete variable genes that were present in all isolates was used as the core genome. To determine if the *N. gonorrhoeae* isolates circulating in remote WA are genetically different from those circulating in the WA metropolitan areas, 72 *N. gonorrhoeae* genomes from diverse international locations (available at PubMLST *Neisseria* spp. isolates database) from a recent epidemiological study by Ezewudo et al. [24] were included. A MAFFT alignment of the concatenated core genome of 1005 genes (excluding the invariant loci of the core genome) was constructed using the genome comparator tool at BIGSdb. Invariant genes were removed to reduce computation load as they will not provide cladistic information [25]. RAxML version 8.0 software was used to create a maximum likelihood core genome phylogenetic tree using the GTRGAMMA model, which combines a GTR (Generalized Time Reversible) model for the rate of substitutions between nucleotides at a site, with a Gamma distribution model for substitution-rate heterogeneity between sites). In this model four discrete rate categories are used providing an acceptable balance between speed and accuracy of analysis. The majority-rule consensus tree was generated from 200 bootstrapped replicates of the model [26]. FigTree software v1.4.2 was used to visualise the generated Newick trees [27]. The TempEst program was used to compute the most likely root for the tree [28]. In a larger scale analysis, the core genome MAFFT alignment (90% core threshold, 2182 genes) of 1053 *N. gonorrhoeae* isolates including the 59 WA and the 72 international *N. gonorrhoeae* isolates was used to construct a neighbor joining phylogeny with 100 bootstrapped replicates of the model using MEGA7: Molecular Evolutionary Genetics Analysis version 7.0 for bigger datasets [29]. These 1053 *N. gonorrhoeae* isolates were selected using maximum variation sampling covering all available MLST STs, locations and years of isolates available at PubMLST database.

### Sequence typing, antimicrobial genetic markers and genomic islands

Using the Short Read Sequence Typing for Bacterial Pathogens (SRST2) software [30, 31]. MLST was performed by comparing the assembled sequences of the seven housekeeping loci (*abcZ, adk, aroE, fumC, gdh, pdhC* and *pgm*) to the reference MLST profiles on the PubMLST database (http://pubmlst.org/neisseria/) [23]. Pileup and scores files generated by SRST2 were used for manual curation. All novel allelic combinations were referred to the PubMLST website curator to be assigned a ST.

NG-MAST was performed on the sequences produced by whole genome sequencing using the genome comparator tool at BIGSdb ((http://pubmlst.org/neisseria/) [32]. The sequences were trimmed, as described in the NG-MAST website (http://www.ng-mast.net/) and submitted to the NG-MAST database for ST determination. An integer was assigned to *porB* (encoding major outer membrane protein porin) and *tbpB* (encoding transferrin binding protein B). All novel alleles were referred to the NG-MAST website curator to be assigned an allelic number and ST.

NG-STAR, a novel molecular antimicrobial resistance typing scheme based on the sequence of seven genes associated with antimicrobial resistance in *N. gonorrhoeae* (*penA*, *mtrR*, *porB*, *ponA*, *gyrA*, *parC* and 23S rRNA) [8], was performed by comparing each allele to the publicly accessible database at https://ngstar.canada.ca by SRST2. All novel alleles and new allele combinations were referred to the NG-STAR curator to be assigned an allelic number and ST.

Plasmids were assembled using SPAdes as single contigs then compared to their corresponding references (β-lactamase producing pJD4 and the gonococcal cryptic pJD1). The variable regions between β-lactamase plasmids were aligned and were assigned based upon the method of Trembizki et al. [33]. The three conjugative plasmids found in *N. gonorrhoeae*, a 39 kb plasmid (pEP5233) (GenBank accession number GU479465.1) and two 42 kb *tetM*-positive, the Dutch (pEP5289) and the American (pEP5050) plasmids (GenBank accession numbers GU479466.1 and GU479464.1) [34] were also identified as single contigs. Prior to plasmid alignment the Cyclic DNA Sequence Aligner software was used to find the optimal rotation for the circular plasmid sequences [35].

The genome comparator tool at PubMLST was used to detect the existence of defined loci of conjugative plasmids and gonococcal genetic islands (GGI) in http://pubmlst.org/neisseria/. The AMR genes 23 S rRNA, *ponA*, *penA*, *porB*, *gyrA*, *parC*, *mtrR*, ^pro^*mtrR*, bla-TEM, *tetM* and *ftsX* were detected using the genome comparator tool and confirmed using SRST2. Novel alleles were aligned and notionally translated to amino acid sequences to enable detection of amino acid substitutions.

### Population structure analysis

Hierarchical clustering of sequences was inferred by using the hierBAPS tandem command line program implemented in BAPS v6.0 to estimate the population structure [36]. Concatenated core genome MAFFT alignments for all 131 *N. gonorrhoeae* isolates in the dataset indicating variable loci that were present in 100% of the isolates in the dataset in FASTA format were used for hierarchical clustering. Clustering was performed with two levels in the hierarchy using *k* = 40 as the prior upper bound for the number of clusters. Deeper levels of clustering was performed based on the first result using *k* = 3 to *k* = 7 K value and the best *k* value in both analyses was six. For the detection and representation of recombination between populations, the hierBAPS output file was converted to BAPS format and the ‘admixture with pre-defined populations’ approach was used in the BAPS software [37].

### Statistical methods

Categorical variables were examined using the Fisher’s Exact test. GraphPad Prism 7 (GraphPad Software Inc., California) was used to perform the analyses. A 5% level of confidence was used and statistical significance was determined with a *p* value of < 0.05. Wallace coefficients measure the extent of congruity between different metrics covering the same data. Adjusted Wallace coefficients (AW) [38] were used to determine the congruence of the three typing methods using the online tool available at http://www.comparingpartitions.info/index.php?link=Tool.

## Results

### Molecular epidemiological typing indicates novel sequence types in remote areas

**Fig. 1:**
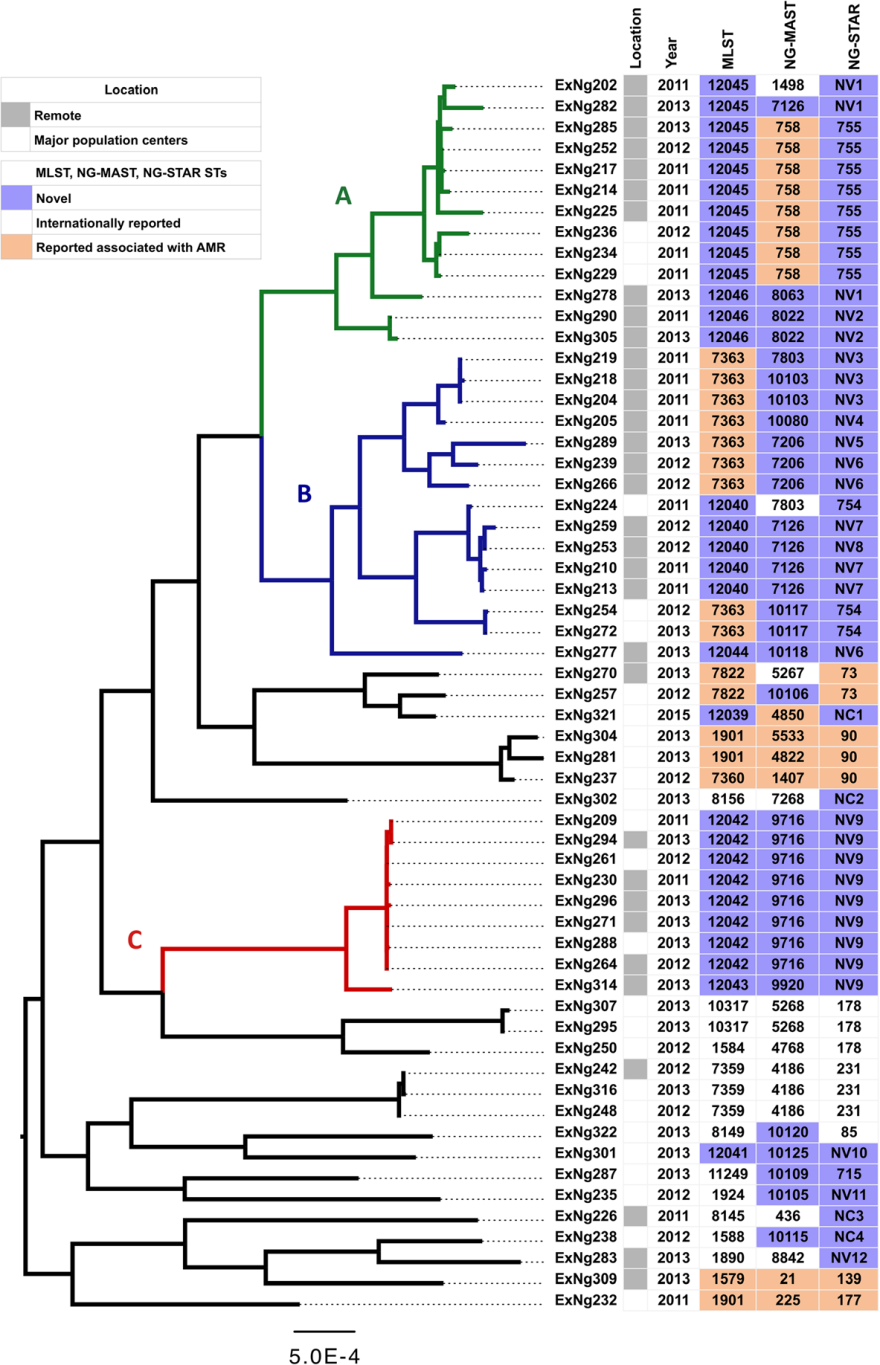
Core genome maximum likelihood phylogeny of 59 strains of *N. gonorrhoeae* from WA. The most likely root for the tree was computed using TempEst [28]. The tree is annotated with geographical location, year of isolation and sequence types derived from three molecular typing methods (MLST, NG-MAST, NG-STAR). Novel and internationally reported sequence types that are associated with AMR are indicated. A, B and C clusters include persistent genetic clusters shared between metropolitan and remote areas of WA

The core genome phylogeny revealed three persistent and stable genetic clusters, clusters A, B and C, which included 37 of the 59 isolates (Fig. 1). Clusters A and B were phylogenetically related and contained 28 isolates of which 78% were from remote WA (*n* = 22, Fig. 1). Cluster C contained 9 isolates of which 67% were from remote WA (*n* = 6). Approximately three quarters (17/22) of the non-A, B, C cluster isolates were collected from metropolitan areas. A significant association (*p* = 0.0001) of the remote isolates with the three clusters A, B and C was observed using Fisher’s exact test.

MLST identified a total of 23 STs, of which eight were new to the international PubMLST database (ST12039–12046), and these eight STs accounted for 51% of isolates (*n* = 30/59) of the collection. The MLST STs found in clusters A (ST12045 and ST12046) and B (ST7363, ST12040 and ST12044) were closely related with isolates sharing five of the seven alleles found in ST7363. Cluster C contained two MLST STs (ST12042 and ST12043) which shared six of the seven alleles. The 22 isolates that did not group into clusters A, B or C shared 17 different MLST STs of which two had not been described before (ST12039, ST12041) [Additional file 2].

NG-MAST typing identified 32 STs. Three quarters of the NG-MAST STs (*n* = 21/32) were singletons. NG-MAST STs with the most isolates were ST758 and ST9716. Both STs were cluster specific, with ST758 making up eight of 13 isolates identified in cluster A and ST9716 making up eight of the nine isolates identified in cluster C [Additional file 2]. The majority of single NG-MAST STs (17/21) were not present in the three clusters.

NG-STAR identified 26 STs. Only seven of the NG-STAR STs have previously been described and these were ST73, ST85, ST90, ST139, ST177, ST178, and ST231. Of the nineteen NG-STAR STs, four had new combinations (NCs) of previously reported alleles and 15 were novel having at least one novel locus (NVs). Table 1 defines all the NC and NV NG-STAR STs identified in this study. NG-STAR NCs STs were not found in clusters A, B and C. The 15 novel NG-STAR STs possessed novel *mtrR* alleles. Three novel profiles have been designated NG-STAR ST715, ST754, and ST755 which have not been described elsewhere. NG-STAR ST755 was only found in cluster A and accounted for 61% (*n* = 8/13) of isolates in this cluster. NG-STAR ST754 characterised three metropolitan isolates in cluster B. In addition, NVs ST11 and ST12 had a novel *gyrA allele*, ST1 had a novel *penA* allele, and ST15 possessed a novel *porB* allele. All isolates in clusters A, B and C had novel NG-STAR STs [Additional file 2].

**Table 1:** NG-STAR Novel (NV) and new allelic combinations (NC) profiles identified in the present study

NG-STAR	*penA*	*mtrR*	*porB*	*ponA*	*gyrA*	*parC*	23S	Isolates associated with the NG-STAR ST
715	19.001	124	0	0	1	20	0	ExNg287
754	2.001	123	14	1	0	1	0	Exng224, ExNg254, ExNg272
755	2.001	123	13	1	0	0	0	ExNg214, ExNg217, ExNg225, ExNg229, ExNg234, ExNg236, ExNg252, ExNg282, ExNg285
NC1	2.002	19	9	1	7	3	1	ExNg321
NC2	63.001	1	0	1	0	2	0	ExNg302
NC4	19.001	47	12	1	7	20	0	ExNg238
NC3	2.001	16	13	0	1	18	0	ExNg226
NV1	2.001	123	14	1	0	0	0	ExNg202, ExNg278, ExNg282
NV2	2.001	123	0	1	0	0	0	ExNg290, ExNg305
NV3	2.001	123	14	1	20	1	0	ExNg204, ExNg218, ExNg219
NV4	2.001	123	0	1	20	1	0	ExNG205
NV5	22.001	123	0	0	0	1	0	ExNg289
NV6	2.001	123	0	1	0	1	0	ExNg239, ExNg266, ExNg277
NV7	2.001	123	14	0	0	1	0	ExNg210, ExNg213, ExNg259
NV8	2.001	123	14	0	0	1	0	ExNg253
NV9	2.002	125	14	1	0	7	0	ExNg209, ExNg230, ExNg261, ExNg264, ExNg271, ExNg288, ExNg294, ExNg296, ExNg314
NV10	22.001	127	3	0	0	7	0	ExNg301
NV11	2.002	54	19	0	0	2	0	ExNG235
NV12	2.008	54	14	1	7	20	0	Exng283

### Population structure analysis identifies two unique cluster groups in Western Australia

**Fig. 2:**
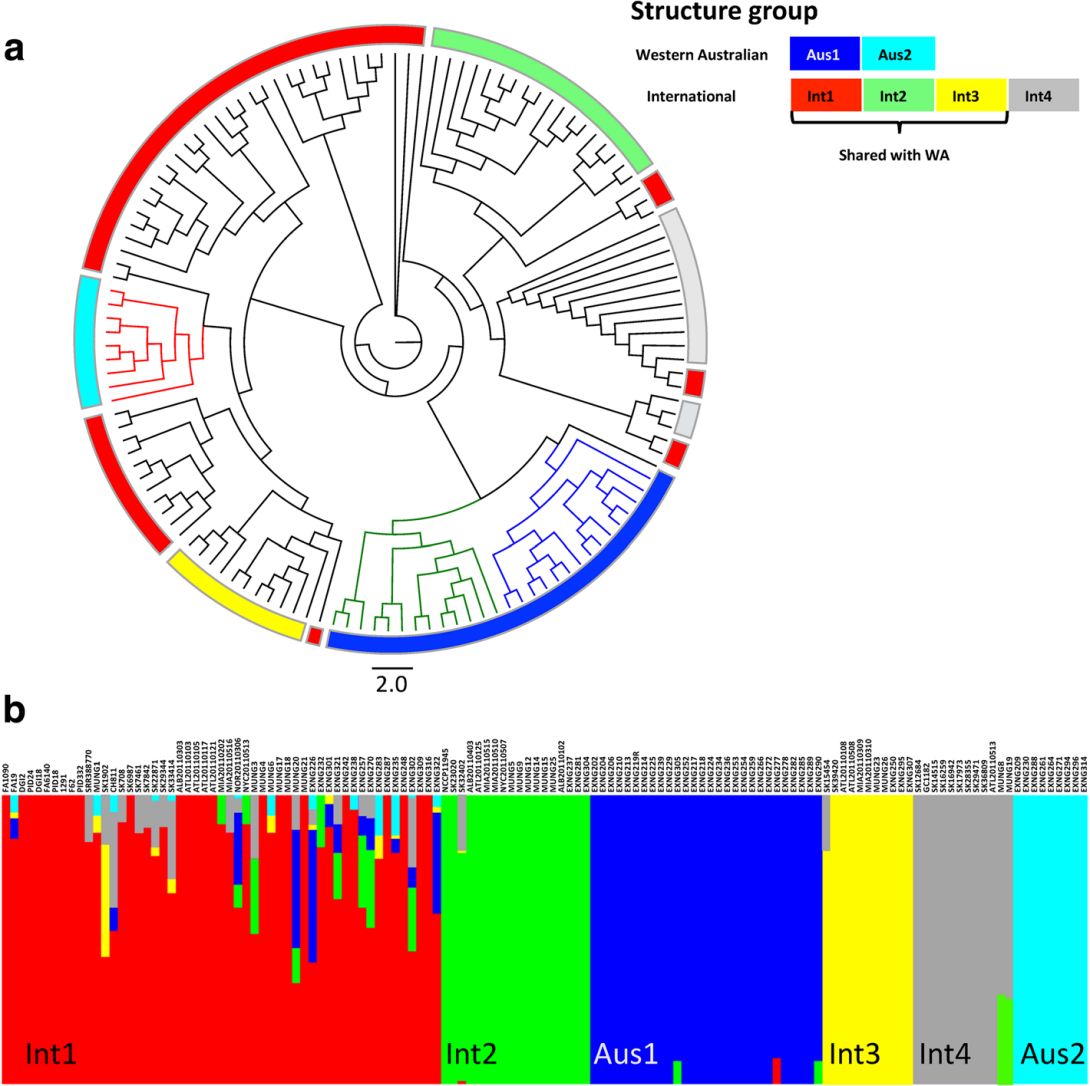
**a** Cladogram of 59 strains of *N. gonorrhoeae* from WA in addition to 72 *N. gonorrhoeae isolates from an international dataset highlighted with* the six hierBAPS defined groups Int1 (red), Int2 (green), Int3 (yellow), Int4 (grey), Aus1 (blue) and Aus2 (cyan) groups*.* **b** *Admixture analysis of population groups from strains of N. gonorrhoeae in the sample set defined by BAPS*. Each vertical bar of color represents an isolate (labelled left to right). The primary color indicates the assignment of the isolate according to heirBAPs as defined in Fig. 2a. When the vertical bar shows two colors, it indicates the proportion of admixture that has occurred between this isolate and other structure groups. *The association of the strains with each population group can be found in* Additional files 2 and 3

Since there were many novel STs in the West Australian dataset, core phylogeny of these isolates was compared to 72 isolates in an international dataset of strains characterized by Ezewudo et al. [24]. The total dataset of 131 *N. gonorrhoeae* isolates formed six genetic groups after structure grouping by heirBAPs (Fig. 2). International isolates appeared in four genetic groups Int1-red, Int2-green, Int3-yellow and Int4-gray. While Ezewudo et al. [24] [Additional file 3] originally identified five structure groups, Groups 3 and 5 from that study which were noted to be highly similar collapsed into one group, Int-1, in this study. Two new groups, Australian Group 1 (Aus1-blue) and Australian Group 2 (Aus2-cyan), were formed of isolates only from the WA dataset.

Int1 was the largest structure group containing 53 isolates representing 35 different MLST STs, distributed among other structure groups in the phylogeny. In this structure group, 16 isolates were from WA of which 68% (*n* = 11) were metropolitan isolates and 62% (*n* = 10) were antibiotic resistant isolates. Int2 contained 15 isolates from the international dataset and 3 isolates from WA dataset with the majority of these being representatives of the MLST ST1901 (*n* = 16). In addition all the WA isolates in Int2 were also NG-STAR ST90 which was the most common ST in the NG-STAR study of Demczuk et al. [8]. Int3 contained 12 isolates in total of which 3 isolates were from metropolitan Perth. The WA isolates in Int3 belonged to NG-STAR ST178 but were closely related to MLST STs that were not associated with AMR. All WA isolates in Int2 and Int3 were isolated from the metropolitan area and contained no novel MLST STs, NG-MAST or NG-STAR STs. Aus1 contained the 28 isolates of clusters A and B and Aus2 contained the nine cluster C isolates. The three clusters also contained most of the isolates with novel MLST, NG-MAST and NG-STAR STs.

The BAPS admixture analysis (Fig. 2b) shows Int1 is characterized by significant recombination and appears to be a nexus for gene exchange with isolates scattered across the phylogenetic tree [24]. Three WA isolates (ExNg242, ExNg248 and ExNg316) from Int1 showed no recombination with other structure groups and were identical by MLST (ST7359), NG-MAST (ST4186) and NG-STAR (ST231) suggesting clonal expansion within the recombinant group. In contrast, isolates from the other structure groups formed tight clusters. Clonal expansion was observed in five hierBAPS groups, most significantly in Aus2, as illustrated by the core genome phylogeny and BAPS admixture analysis. Based on adjusted Wallace coefficients (AW), the isolates grouped by MLST have a 97% chance of grouping by hierBAPS, 53% by NG-MAST and 42% by NG-STAR.

**Fig. 3:**
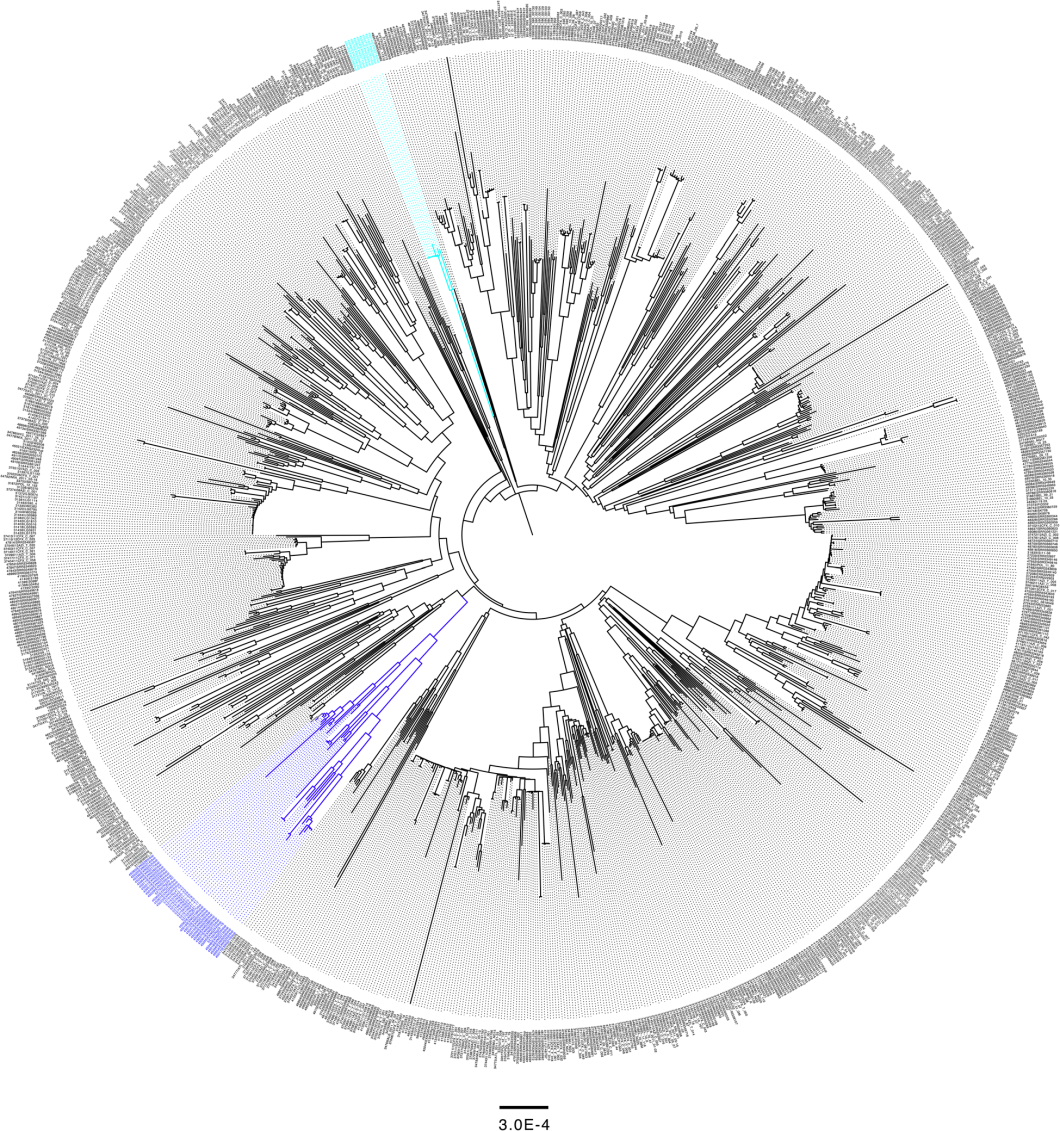
Neighbor joining tree generated with 100 bootstrapped replicates of 1053 *N. gonorrhoeae* isolates from PubMLST database. The tree is highlighted with Australian structure Groups Aus1 (blue) and Aus2 (cyan). Further information associated with all 1053 isolates can be accessed using these tags in the Bacterial Isolates Genome Sequence database (BIGSdb) genomics platform (www.pubmlst.org/neisseria)

To further validate the clusters identified by heirBAPs using the 131 strain dataset, a Neighbor joining tree was constructed using the core genome of 1053 *N. gonorrhoeae* isolates sourced from PubMLST. Australian structure groups (Aus1 and Aus2) still formed two unique clusters with Aus1 being supplemented with 11 isolates from Queensland (Australia) (Fig. 3).

### Australian structure groups are characterized by an absence of antibiotic resistance and the presence of the gonococcal genetic island

**Fig. 4:**
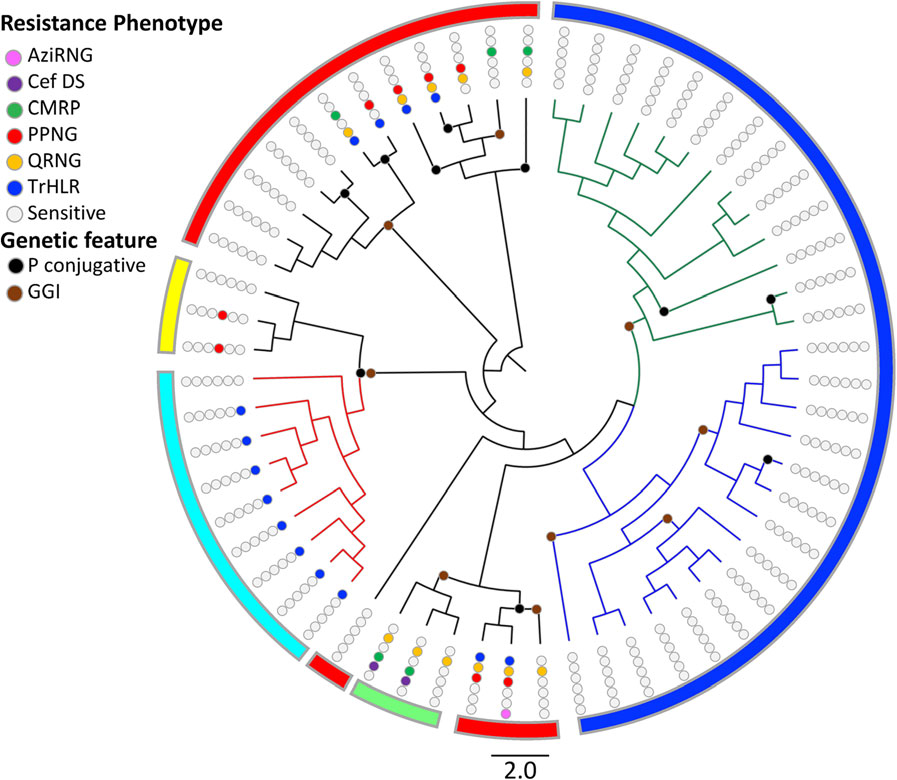
Antibiotic resistance profiles and genetic features of *N. gonorrhoeae* WA strains across population groups. A radial representation of the phylogenetic tree from Fig. 1 showing the antibiotic resistance profiles and genetic features. The outer line color reflects hierBAPS defined groups. (Cef DS) ceftriaxone decreased susceptibility, (AziRNG) azithromycin-resistant *N. gonorrhoeae*, (TrHLR) high-level resistance to tetracycline, (PPNG) Penicillinase-producing *N. gonorrhoeae*, (CMRP) Chromosomal mediated resistance to penicillin, (QRNG) quinolone-resistant *N. gonorrhoeae,* (P conjugative) conjugative plasmid, (GGI) Gonococcal genetic island. Alleles associated with these phenotypes are presented in Additional file 2

The association between the genetic groups, AMR and selected genomic features of the 59 WA isolates were analyzed. The gonococcal genetic island (GGI) was identified in 86% (50/59) of isolates (Fig. 4). Conjugative plasmids, which often carry the *tetM* determinant, were present in 42% (24/59) of the isolates. The markerless 39 kb plasmid (pEP5233) was found in two Int1, three Int3 and three Aus1 isolates. pEP5289 (Dutch), which carries *tetM*, was found in five Int1 tetracycline HLR isolates. pEP5050 (American) was found in all Aus2 isolates and two Int1 isolates, and apart from ExNg314 all isolates were tetracycline HLR. Two previously identified beta-lactamase plasmid types, African (pJD5) and Rio/Torino plasmid (pJD7) were found in seven Int1 and two Int3 penicillinase-producing *N. gonorrhoeae* (PPNG) isolates [Additional file 2].

Other than tetracycline resistance in Aus2, all Aus1 and Aus2 isolates did not show resistance to beta-lactams, quinolones or macrolides (Fig. 4) and lacked previously reported mutations associated with these antibiotics (Additional file 2). However, the Aus1 and Aus2 isolates did possess an A39T mutation in the coding sequence of the repressor gene (*mtrR,* NEIS1635) which has been shown to result in the over-expression of the MtrCDE efflux pump system but not with wild type *mtrR* promoter [39].

Twenty-two WA isolates clustered in Int1, Int2 and Int3 structure groups. Approximately two thirds (62%, 16/22) were resistant to one or more antibiotics. A *penA* type 63 mosaic allele (NEIS1753 allele 1147) was identified in one susceptible strain from Int1. Int2 isolates contained NG-STAR ST90 that has been reported associated with ESCs and ciprofloxacin resistance. Decreased susceptibility to the ESCs due to the *penA* (NEIS1753) motif XXXIV was restricted to Int2. Int2 isolates also contained GGI and (pEP5233) conjugative plasmids. Int3 did not contain any chromosomal mutations affecting beta-lactamase, quinolone and macrolide susceptibility consistent with the antibiotic sensitive phenotype. *mtrR* with an internal stop codon but no A39T or G45D mutations was found in all Int3 isolates [Additional file 2].

All isolates with high-level resistance to ciprofloxacin (QRNG HLR) had mutations in *gyrA* (NEIS1320) and *parC,* (NEIS1525) conferring resistance to quinolones. High-level resistance to azithromycin (AziRNG HLR) mediated by the A2059G mutation in genes encoding for 23S rRNA was identified in AziRNG HLR isolate ExNg321. A recently reported R251H substitution in the *ftsX* gene (NEIS2146 allele 18) which confers ESCs resistance [40, 41], was found in five extra-genital *N. gonorrhoeae* isolates however only two of these isolates demonstrated decreased susceptibility to ceftriaxone.

## Discussion

The high rate of recombination within the *N. gonorrhoeae* population has led to the presumption that this species has a non-clonal population structure [42]. However, clonal spread and persistence of *N. gonorrhoeae* strains has been described in many whole genome sequence-based epidemiological studies from Europe, France, USA, Canada, and recently from a global collection of *N. gonorrhoeae* [11, 12, 14, 15, 43]. Population structure analysis of *N. gonorrhoeae* isolates in the study dataset has provided some evidence for both recombination and clonality in *N. gonorrhoeae*, suggesting *N. gonorrhoeae* is a weakly clonal species similar to *H. pylori* and *Bacillus sphaericus* [12, 44, 45]. However, an analysis of an expanded dataset over a prolonged period of time would provide a much clearer understanding of the *N. gonorrhoeae* population structure.

In the remote areas of WA, when compared to the global population, gonococcal isolates formed two distinctive structure groups (Aus1 and Aus2). Aus1 was characterized by broad antibiotic-susceptibility and was mostly composed of isolates related to novel MLST, NG-MAST and NG-STAR STs. The novelty of the MLST, NG-MAST ST and NG-STAR designations is significant as the isolates were compared to over 3700 isolates found in the PubMLST database, which represents collections from around the world, particularly Europe and the US. All isolates in Aus1 belonged to two core genome clusters, A and B. Isolates belonging to MLST ST7367, which have been reported from global sources, are associated with decreased susceptibility to ceftriaxone and have been shown to cluster with Int1 by Ezewudo et al. [24]. However, the MLST ST7363 isolates of Aus1 (cluster A) formed a discrete cluster group apart from Int1, suggesting they are unique. In cluster A of Aus1, MLST ST12045 isolates were associated with a previously reported NG-MAST ST758 reported from Russia which is associated with decreased susceptibility to ceftriaxone [46]. However, the Aus1 MLST ST12045 isolates were susceptible to ceftriaxone and possessed novel NG-STAR profiles. Collectively this suggests Aus1 is a distinctive population of isolates circulating in remote Australian regions. Aus2 represents a local dissemination of a highly clonal tetracycline HLR population which was characterized by novel MLST, NG-MAST and NG-STAR assignments. The presence of pEP5050 (American) conjugative plasmid in Aus2 suggests a global introduction of the initial strain to the region followed by clonal persistence of this group. One Aus2 isolate that was not tetracycline HLR, ExNg314, possessed an intact conjugative plasmid carrying the *tetM* determinant but did not have the V57 M in *rspJ* gene, unlike the other tetracycline HLR isolates in this group [47]. This suggests the V57 M mutation in combination with the plasmid mediated resistance determinant is required to achieve high-level tetracycline resistance. The absence of other mutations in the AMR genetic markers in the Aus groups suggests no independent emergence of resistance (de novo) since divergence from its ancestor. Lastly Aus1 and Aus2 were characterized by possession of *porB1a* (68%, 27/40) which is associated with asymptomatic urethral infections and disseminated gonococcal infection (DGI) [48, 49]. Though *porB1a* is not associated with MDR strains, monitoring these isolates is crucial as DGI is considered a common cause of complicated gonococcal infection and infertility [49, 50].

Apart from Aus1 and Aus2, 22/59 isolates from WA in this period clustered with the Int1, Int2 and Int3 structure groups. WA isolates from Int1 were retrieved primarily from metropolitan areas. The strains are genetically unrelated and highly recombinant, and suggest the sporadic emergence of various *N. gonorrhoeae* clusters or represent sporadic incursions of international strains into WA. The ciprofloxacin resistant isolate ExNg232 which had a NG-MAST 225 and NG-STAR177 STs, was previously reported in Europe and the US [11, 12], suggesting an introduction event to the metropolitan areas of WA. In another instance, a high-level azithromycin resistant isolate (ExNg321) had a NG-MAST ST4850 which differed by one SNP from a ST1866 AziRNG HLR isolate reported in China and a MLST ST12039 that was a single locus variant of a ST10899 AziRNG HLR isolate reported in Canada [24, 51]. This would suggest that ExNg321 is a foreign introduction into WA and may be one of the earliest isolates to cause a recent outbreak of azithromycin resistant strains in South Australia that led to an updated treatment recommendation to avoid single agent azithromycin treatment [52]. The antibiotic sensitive isolates of Int1 from MLST7359 have recently been linked to increasing rates of gonococcal infections among women in heterosexual networks in metropolitan areas of New South Wales in Australia [53].

Three isolates that clustered with Int2 represented the global AMR cluster MLST ST1901 and the two related STs (matching at six MLST loci) that are associated with decreased susceptibility and resistance to ceftriaxone [11, 12, 41]. One of the isolates (ExNg304) belonged to NG-STAR ST90, which has been reported to be associated with decreased susceptibility and resistance to ceftriaxone and ciprofloxacin. However, although ExNg304 had all the requisite mutations for this phenotype, the isolate was phenotypically susceptible. Lastly, all the WA isolates in Int3 were NG-STAR ST178, a cluster that is antibiotic susceptible and does not contain any MLST or NG-MAST STs associated with resistance [8]. This appears to represent a potential persistent susceptible cluster that has been circulating globally since 1998 [24].

The gonococcal genetic island (GGI) reported by Harrison et al. [54] has been significantly associated with different MDR core genome clusters such as clusters of MLST ST1901 and ST1508. In the present study, GGI was identified in most isolates (50/59, 84%) in WA *N. gonorrhoeae* populations, being found in 68% (13/19) of isolates in MDR groups Int1 and Int2 and 95% (38/40) of isolates in the non-MDR groups Aus1, Aus2 and Int3. This suggests that the presence of GGI in a given structure group may provide a fitness advantage and contribute to the stability of that cluster, which occurred before the acquisition of the MDR loci by natural transformation or mutation.

## Conclusions

The population structure of a small set of gonococcal isolates from WA has revealed the presence of two unique clusters: Aus1 and Aus2. Due to the small sample size and the lack of representative isolates from Australia and SouthEast Asia in the PubMLST database, it remains to be determined if these two clusters are more widely dispersed in this geographic region. However, the clustering of 11 gonococcal isolates from Queensland into Aus1 is suggestive of a broader distribution across Australia which will be confirmed as much larger studies in these regions are underway. The persistence of the antibiotic susceptible cluster Aus1 in WA could be due to azithromycin and amoxicillin dual therapy in remote WA, thereby reducing the selective pressure for AMR or could be the result of the remoteness of this region which has impeded incursion of AMR strains [19]. The NG-STAR typing scheme correlates well with core genome phylogeny of *N. gonorrhoeae* and is sufficient for high throughput surveillance of AMR. However with the recent report that Bexsero^®^ vaccination may reduce the rate of gonorrhoea [55], WGS is needed for antigenic profiling and monitoring the persistence of antigenic combinations over short timeframes in response to vaccination in clinical trials and given its high level of discrimination is the most accurate way of defining sexual networks.

## Additional files

Additional file 1: **Table S1.** Sequencing and assembly quality statistics of the 59 Western Australian *N. gonorrhoeae* isolates. (PDF 63 kb)

Additional file 2: **Table S2.** WA *N. gonorrhoeae* isolates metadata, antimicrobial resistance profiles, sequence types and genetic characteristics of antimicrobial resistance markers. (PDF 95 kb)

Additional file 3: **Table S3.** List of the 72 international *N. gonorrhoeae* isolates from Ezewudo et al. [24]. (PDF 72 kb)

## Abbreviations

AMR: Antimicrobial resistance
Aus1: Australian group1
Aus2: Australian group 2
AziRNG: Azithromycin-resistant *N. gonorrhoeae*
BIGSdb: Bacterial Isolates Genome Sequence database
DGI: Disseminated gonococcal infection
ESCs: Extended-spectrum cephalosporins
GASP: Gonococcal Antimicrobial Surveillance Programme
GGI: Gonococcal genetic island
HLR: High-level resistance
MDR: Multi-drug resistant
MIC: Minimum inhibitory concentration
MLST: Multilocus sequence typing
MSM: Men who have sex with men
NC: New combination
NG-MAST: *N. gonorrhoeae* multi antigen sequence typing
NG-STAR: *N. gonorrhoeae* sequence typing for antimicrobial resistance
NT: Northern Territory
NV: Novel
PPNG: Penicillinase-producing *N. gonorrhoeae*
QRNG: Quinolone-resistant *N. gonorrhoeae*
ST: Sequence type
WA: Western Australia
WAGSP: Western Australian Gonococcal Surveillance Programme
WGS: Whole genome sequencing
