# Supplementary material for: Genomic epidemiology and population structure of Neisseria gonorrhoeae from remote highly endemic Western Australian populations
Source: BMC Genomics. 2018 Feb 27;19:165. doi: 10.1186/s12864-018-4557-5 (PMC6889462; doi:10.1186/s12864-018-4557-5)
Supplement: Supplementary file 1 — Table S1. Sequencing and assembly quality statistics of the 59 Western Australian N. gonorrhoeae isolates. (PDF 63 kb) [file 12864_2018_4557_MOESM1_ESM.pdf]

Table S1: Sequencing and assembly quality statistics of the 59 Western Australian *N. gonorrhoeae* isolates

ISOLATE	NUMBER READS	MAXIMUM READ LENGTH	Total length (>= 0 bp)	Total length (>= 1000 bp)	# contigs	Largest contig	Total length	GC (%)	N50	N75	L50	L75
EXNG202	1650072	301	2210215	2148505	159	112181	2177922	52.37	38575	18981	18	39
EXNG204	1402770	301	2173781	2151201	90	205090	2157908	52.43	52649	30902	11	24
EXNG205	1278354	301	2199580	2151855	137	113649	2166323	52.48	41022	20363	19	37
EXNG209	1700186	301	2230161	2192634	103	189628	2206292	52.33	60181	32523	12	24
EXNG210	1110594	301	2164541	2137523	132	123865	2148368	52.51	31701	17564	18	41
EXNG213	1457780	301	2190869	2143572	126	167663	2164345	52.27	39671	20763	14	32
EXNG214	1530790	301	2173642	2141494	101	170009	2151308	52.48	55228	20720	12	27
EXNG217	2498630	301	2187923	2143945	106	243068	2156314	52.43	49895	24489	11	25
EXNG218	1395016	301	2174053	2145529	125	170374	2156256	52.44	47349	19878	14	33
EXNG219	1075998	301	2168334	2145743	122	109169	2159835	52.53	45743	20470	18	35
EXNG224	1959886	301	2159483	2132550	95	262884	2143041	52.47	57806	26499	9	23
EXNG225	1838888	301	2194441	2145733	125	104207	2159207	52.49	32790	19492	20	41
EXNG226	1510216	301	2189852	2145569	130	131577	2168199	52.42	41168	21725	16	34
EXNG229	915446	301	2202234	2143355	144	189697	2162298	52.5	45562	20185	16	35
EXNG230	1634624	301	2224339	2192639	111	207691	2213645	52.28	61143	28102	10	23
EXNG232	2016894	301	2144223	2092595	123	175521	2115087	52.66	57450	25832	13	27
EXNG234	1329232	301	2187913	2149986	94	192498	2164232	52.41	64443	30077	11	23
EXNG235	1788934	301	2220434	2193801	94	173743	2208121	52.37	61761	31628	9	22
EXNG236	1630616	301	2154083	2119147	144	101077	2132786	52.64	30976	17172	21	44
EXNG237	748372	301	2179678	2153821	100	113506	2167147	52.37	59250	27048	15	27
EXNG238	813842	301	2150724	2131293	113	140722	2140967	52.6	40610	23054	17	35
EXNG239	1206320	301	2179359	2154361	108	243099	2167213	52.38	55670	23540	12	26
EXNG242	1695488	301	2147235	2119974	96	1133987	2130736	52.59	46083	23523	15	31
EXNG248	1256682	301	2174053	2137503	115	101873	2150979	52.58	41204	25180	17	33
EXNG250	1268026	301	2190449	2171015	96	175984	2178083	52.53	48117	22998	13	29
EXNG252	1463466	301	2161090	2138917	89	205101	2145291	52.48	52964	23864	12	27
EXNG253	1444344	301	2177510	2144153	113	308494	2158734	52.46	49697	23360	11	26
EXNG254	1389218	301	2106497	2088950	94	126447	2093615	52.75	57398	20877	14	29
EXNG257	990974	301	2167302	2129515	150	133188	2144947	52.66	31730	18203	20	45
EXNG259	1324310	301	2193243	2133764	152	177791	2160359	52.36	38679	18865	18	39
EXNG261	1231512	301	2219973	2191248	98	233417	2202964	52.42	55945	30443	12	26
EXNG264	1685018	301	2247459	2183393	124	207513	2207258	52.2	47369	24693	14	30
EXNG266	1499634	301	2161538	2137871	107	127931	2148444	52.54	39371	21535	18	36
EXNG270	1317404	301	2205242	2184299	99	173901	2196995	52.41	57422	31642	13	25
EXNG271	2538250	301	2217722	2181724	88	207508	2191366	52.36	60596	32296	11	23
EXNG272	1376266	301	2124225	2098724	95	238336	2110249	52.66	59669	27518	10	23
EXNG277	1893236	301	2166508	2137790	103	197688	2149676	52.46	53465	23491	11	26
EXNG278	1670082	301	2218816	2183072	119	205495	2200387	52.25	45668	23471	14	30
EXNG281	1114386	301	2175034	2151153	89	207853	2158576	52.39	52970	31591	10	23
EXNG282	1093340	301	2194481	2155929	86	243051	2163834	52.4	64590	41165	10	20
EXNG283	1310872	301	2110473	2081088	112	139005	2089661	52.72	41082	24285	17	34
EXNG285	2026754	301	2186048	2146836	92	243141	2157082	52.46	57647	30409	11	23
EXNG287	783198	301	2218700	2185760	159	99326	2207943	52.43	36802	16537	21	45
EXNG288	1625042	301	2222159	2185404	99	178451	2196932	52.4	47153	23861	14	30
EXNG289	1063504	301	2213293	2178848	136	113312	2198039	52.41	39101	19270	17	38
EXNG290	1659254	301	2230374	2198376	100	170075	2209491	52.32	63784	31128	11	25
EXNG294	957682	301	2223597	2181460	111	189688	2192944	52.39	43017	21758	13	31
EXNG295	1081510	301	2174040	2147622	140	90722	2165339	52.57	32496	18118	22	45
EXNG296	1384290	301	2223512	2187862	102	207514	2199216	52.32	47025	27893	13	27
EXNG301	2160786	301	2303774	2205909	157	203345	2249084	52.33	54915	25524	12	28
EXNG302	1432752	301	2139201	2090855	99	207686	2104526	52.74	59527	25770	11	24
EXNG304	1236338	301	2165226	2145511	88	207781	2153703	52.43	59316	32046	10	23
EXNG305	1339424	301	2186673	2139619	124	146586	2154048	52.5	46950	20815	15	32
EXNG307	1314822	301	2186273	2171368	79	207914	2177591	52.43	57800	35166	11	23
EXNG309	1581012	301	2161813	2147706	70	216269	2150766	52.52	78145	39072	8	18
EXNG314	1583528	301	2326137	2224233	213	194342	2277597	52.35	37349	16845	19	43
EXNG316	803852	301	2154234	2136579	102	134110	2146754	52.57	49281	26531	16	31
EXNG321	627910	301	2154273	2130559	130	96793	2143589	52.62	35329	18758	19	41
EXNG322	2932636	301	2232748	2209637	100	208429	2219751	52.32	46712	29586	14	28

N50 The shortest sequence length at 50% of the genome
N75 The shortest sequence length at 75% of the genome
L50 The smallest number of contigs whose length sum produces N50
L75 The smallest number of contigs whose length sum produces N75
