# Supplementary material for: Genomic epidemiology and population structure of Neisseria gonorrhoeae from remote highly endemic Western Australian populations
Source: BMC Genomics. 2018 Feb 27;19:165. doi: 10.1186/s12864-018-4557-5 (PMC6889462; doi:10.1186/s12864-018-4557-5)
Supplement: Supplementary file 2 — Table S2. WA N. gonorrhoeae isolates metadata, antimicrobial resistance profiles, sequence types and genetic characteristics of antimicrobial resistance markers. (PDF 95 kb) [file 12864_2018_4557_MOESM2_ESM.pdf]

Table S2: Western Australian *N. gonorrhoea* e isolates metadata and genetic characteristics of antimicrobial resistance

																				Macrolids	B-lactams	B-lactams	B-lactams	B-lactams	Fluroqinolones	Fluroqinolones	Efflux pumps	Efflux pumps	plasmid TetM	plasmid b-lactams	ESCs		
**Isolate**	**RecdDate**	**Location**	**Pen MIC**	**Pen Interpretation**	**Cef MIC**	**Cef Interpretation**	**Cip MIC**	**Cip Interpretation**	**Tet MIC**	**Tet Interpretation**	**Spe MIC**	**Azi MIC**	**Azi Interpretation**	**Spe Interpretation**	**Core genome cluster**	**Population structure group**	**MLST**	**NG-MAST**	**NG-STAR**	23S_rRNA	NEIS0414 *ponA*	NEIS1753 *penA*	NEIS2020 *porB*	*porB* type	NEIS1320 *gyrA*	NEIS1525 *parC*	NEIS1635 *mtrR*	pro_NEIS1635	NEIS2210	NEIS2357	NEIS2146 *ftsX*	GGI	C plasmid backbone
ExNg 202	1/18/2011	Remote	0.125	LS	<=0.008	S	<=0.03	S	<=8	Not TRNG	<=64	<=0.5	S	S	A	Aus1	12045	1498	NV17	10	13 (L421P)	166	1429	1A	230	952	812 (A39T)	6	Neg	Neg	612	Pos	Neg
ExNg 204	1/22/2011	Remote	0.25	LS	<=0.008	S	<=0.03	S	<=8	Not TRNG	<=64	<=0.5	S	S	B	Aus1	7363	10103	NV12	10	13 (L421P)	166	704	1A	807	402	808 (A39T)	6	Neg	Neg	17	Pos	Neg
ExNg 205	2/2/2011	Remote	0.25	LS	<=0.008	S	<=0.03	S	<=8	Not TRNG	<=64	<=0.5	S	S	B	Aus1	7363	10080	NV11	10	13 (L421P)	166	1430 (G120N, A121V)	1B	807	402	808 (A39T)	6	Neg	Neg	17	Pos	Neg
ExNg 209	3/11/2011	Metropolitan	0.125	LS	<=0.008	S	<=0.03	S	**>=16**	**TRNG**	<=64	<=0.5	S	S	C	Aus2	12042	9716	NV5	539	13 (L421P)	23	1431	1A	19	951	953 (ISC)	6	2 (American)	Neg	17	Pos	pEp5050 (American)
ExNg 210	3/28/2011	Remote	0.06	LS	<=0.008	S	<=0.03	S	<=8	Not TRNG	<=64	<=0.6	S	S	B	Aus1	12040	7126	NV8	10	8	166	704	1A	230	402	808 (A39T)	6	Neg	Neg	17	Pos	Nge
ExNg 213	5/19/2011	Remote	0.125	LS	0.016	S	<=0.03	S	<=8	Not TRNG	<=64	<=0.5	S	S	B	Aus1	12040	7126	NV8	10	8	166	704	1A	230	402	808 (A39T)	6	Neg	Neg	17	Pos	Nge
ExNg 214	6/1/2011	Remote	0.125	LS	<=0.008	S	<=0.03	S	<=8	Not TRNG	<=64	<=0.5	S	S	A	Aus1	12045	758	NV15	10	13 (L421P)	166	536	1A	230	955	951 (ISC)	6	Neg	Neg	612	Pos	Neg
ExNg 217	6/15/2011	Remote	0.125	LS	<=0.008	S	<=0.03	S	<=8	Not TRNG	<=64	<=0.5	S	S	A	Aus1	12045	758	NV14	10	13 (L421P)	166	536	1A	230	955	808 (A39T)	6	Neg	Neg	612	Pos	Neg
ExNg 218	6/27/2011	Remote	0.06	LS	<=0.008	S	<=0.03	S	<=8	Not TRNG	<=64	<=0.5	S	S	B	Aus1	7363	10103	NV12	10	13 (L421P)	166	704	1A	807	402	808 (A39T)	6	Neg	Neg	17	Pos	Neg
ExNg 219	7/4/2011	Remote	0.125	LS	<=0.008	S	<=0.03	S	<=8	Not TRNG	<=64	<=0.5	S	S	B	Aus1	7363	7803	NV12	10	13 (L421P)	166	704	1A	807	402	808 (A39T)	6	Neg	Neg	17	Pos	Neg
ExNg 224	7/26/2011	Metropolitan	0.25	LS	<=0.008	S	<=0.03	S	<=8	Not TRNG	<=64	<=0.5	S	S	B	Aus1	12040	7803	NV7	10	13 (L421P)	166	669	1A	230	402	808 (A39T)	6	Neg	Neg	17	Pos	Nge
ExNg 225	8/3/2011	Remote	0.125	LS	<=0.008	S	<=0.03	S	<=8	Not TRNG	<=64	<=0.5	S	S	A	Aus1	12045	758	NV14	10	13 (L421P)	166	536	1A	230	953	808 (A39T)	6	Neg	Neg	612	Pos	Neg
ExNg 226	9/5/2011	Remote	**>2**	**PPNG**	<=0.008	S	**1-2**	**R**	**>=16**	**TRNG**	<=64	<=0.5	S	S		Int1	8145	436	NC2	430	8	166	1432	1A	234 (S91F, D95A)	948 (D86N)	16	3 (−35A)	1 (Dutch)	3 (blaTEM-1)	17	Neg	pEp5050 (American)
ExNg 229	11/15/2011	Metropolitan	0.125	LS	<=0.008	S	<=0.03	S	<=8	Not TRNG	<=64	<=0.5	S	S	A	Aus1	12045	758	NV14	10	13 (L421P)	166	536	1A	230	952	808 (A39T)	6	Neg	Neg	17	Pos	Neg
ExNg 230	11/15/2011	Remote	0.125	LS	<=0.008	S	<=0.03	S	**>=16**	**TRNG**	<=64	<=0.5	S	S	C	Aus2	12042	9716	NV5	539	13 (L421P)	23	1431	1A	19	951	953 (ISC)	6	2 (American)	Neg	17	Pos	pEp5050 (American)
ExNg 232	11/17/2011	Metropolitan	**1**	**CMRP**	0.016	S	**>=4**	**HLR**	<=8	Not TRNG	<=64	<=0.5	S	S		Int1	1901	225	177	231	13 (L421P)	283	544 (G120K, A121D)	1B	231 (S91F, D95G)	243 (S87R)	13	3 (−35A)	Neg	Neg	18 (R251H)	Neg	Neg
ExNg 234	11/28/2011	Metropolitan	0.125	LS	<=0.008	S	<=0.03	S	<=8	Not TRNG	<=64	<=0.5	S	S	A	Aus1	12045	758	NV14	10	13 (L421P)	166	536	1A	230	952	808 (A39T)	6	Neg	Neg	17	Pos	Neg
ExNg 235	1/3/2012	Metropolitan	**1**	**PPNG**	<=0.008	S	<=0.03	S	**>=16**	**TRNG**	<=64	<=0.5	S	S		Int1	1924	10105	NV2	234	8	23	1441	1A	19	631	193 (A39T)	6	1 (Dutch)	3 (blaTEM-1)	20	Pos	pEP5289 (Dutch)
ExNg 236	1/6/2012	Metropolitan	0.125	LS	<=0.008	S	<=0.03	S	<=8	Not TRNG	<=64	<=0.5	S	S	A	Aus1	12045	758	NV14	10	13 (L421P)	166	536	1A	230	952	808 (A39T)	6	Neg	Neg	17	Pos	Neg
ExNg 237	2/7/2012	Metropolitan	**1**	**CMRP**	**0.06**	**DS**	**>=4**	**HLR**	<=8	Not TRNG	<=64	<=0.5	S	S		Int2	7360	1407	90	387	17 (L421P)	266 (XXXIV Mosaic)	552 (G120K, A121N)	1B	14 (S91F, D95G)	104 (S87R)	13	3 (−35A)	Neg	Neg	18 (R251H)	Pos	Neg
ExNg 238	2/18/2012	Metropolitan	**>2**	**PPNG**	<=0.008	S	**>=4**	**HLR**	**>=16**	**TRNG**	<=64	<=0.5	S	S		Int1	1588	10115	NC1	231	13 (L421P)	228	1436 (G120K, A121G)	1B	234 (S91F, D95A)	405 (S87N)	333 (A39T)	6	2 (American)	3 (blaTEM-1)	17	Neg	pEp5050 (American)
ExNg 239	2/27/2012	Remote	0.25	LS	<=0.008	S	<=0.03	S	<=8	Not TRNG	<=64	<=0.5	S	S	B	Aus1	7363	7206	NV6	10	13 (L421P)	166	1430 (G120N, A121V)	1B	230	947	808 (A39T)	6	Neg	Neg	20	Pos	Nge
ExNg 242	2/28/2012	Remote	0.25	LS	<=0.008	S	<=0.03	S	<=8	Not TRNG	<=64	<=0.5	S	S		Int1	7359	4186	231	231	607	23	1435 (G120N, A121V)	1B	646	804	193 (A39T)	14	Neg	Neg	20	Pos	Neg
ExNg 248	5/16/2012	Metropolitan	0.125	LS	<=0.008	S	<=0.03	S	<=8	Not TRNG	<=64	<=0.5	S	S		Int1	7359	4186	231	231	607	23	1435 (G120N, A121V)	1B	646	804	193 (A39T)	14	Neg	Neg	20	Pos	Neg
ExNg 250	6/28/2012	Metropolitan	0.125	LS	<=0.008	S	<=0.03	S	<=8	Not TRNG	<=64	<=0.5	S	S		Int3	1584	4768	178	231	8	294	874 (G120N, A121V)	1B	19	260	371 (ISC)	6	Neg	Neg	17	Pos	pEP5233
ExNg 252	7/23/2012	Remote	0.125	LS	<=0.008	S	<=0.03	S	<=8	Not TRNG	<=64	<=0.5	S	S	A	Aus1	12045	758	NV14	538	13 (L421P)	166	536	1A	230	952	809 (A39T)	6	Neg	Neg	612	Pos	Neg
ExNg 253	7/30/2012	Remote	0.125	LS	<=0.008	S	<=0.03	S	<=8	Not TRNG	<=64	<=0.5	S	S	B	Aus1	12040	7126	NV9	10	8	166	704	1A	230	402	808 (A39T)	6	Neg	Neg	17	Pos	Nge
ExNg 254	8/21/2012	Metropolitan	0.25	LS	<=0.008	S	<=0.03	S	<=8	Not TRNG	<=64	<=0.5	S	S	B	Aus1	7363	10117	NV7	10	13 (L421P)	166	1442	1A	230	402	808 (A39T)	6	Neg	Neg	17	Neg	Nge
ExNg 257	9/4/2012	Metropolitan	**>2**	**PPNG**	0.016	S	**>=4**	**HLR**	**>=16**	**TRNG**	<=64	<=0.5	S	S		Int1	7822	10106	73	430	15 (L421P)	23	1241(G120K, A121D)	1B	234 (S91F, D95A)	509 (S87R)	12 (G45D)	3 (−35A)	1 (Dutch)	2 (blaTEM-135)	17	Neg	pEP5289 (Dutch)
ExNg 259	9/21/2012	Remote	0.125	LS	<=0.008	S	<=0.03	S	<=8	Not TRNG	<=64	<=0.5	S	S	B	Aus1	12040	7126	NV8	10	8	166	704	1A	230	402	808 (A39T)	6	Neg	Neg	17	Pos	Nge
ExNg 261	9/29/2012	Metropolitan	0.125	LS	<=0.008	S	<=0.03	S	**>=16**	**TRNG**	<=64	<=0.5	S	S	C	Aus2	12042	9716	NV5	539	13 (L421P)	23	1431	1A	19	951	953 (ISC)	6	2 (American)	Neg	17	Pos	pEp5050 (American)
ExNg 264	10/8/2012	Remote	0.25	LS	<=0.008	S	<=0.03	S	**>=16**	**TRNG**	<=64	<=0.5	S	S	C	Aus2	12042	9716	NV5	539	13 (L421P)	23	1431	1A	19	951	953 (ISC)	6	2 (American)	Neg	17	Pos	pEp5050 (American)
ExNg 266	10/9/2012	Remote	0.25	LS	<=0.008	S	<=0.03	S	<=8	Not TRNG	<=64	<=0.5	S	S	B	Aus1	7363	7206	NV6	10	13 (L421P)	166	1430 (G120N, A121V)	1B	816	947	808 (A39T)	6	Neg	Neg	20	Pos	Nge
ExNg 270	1/10/2013	Remote	0.25	LS	<=0.008	S	**>=4**	**HLR**	<=8	Not TRNG	<=64	<=0.5	S	S		Int1	7822	5267	73	504	16 (L421P)	23	1437 (G120K, A121D)	1B	234 (S91F, D95A)	509 (S87R)	12 (G45D)	3 (−35A)	new (ISC)	Neg	17	Pos	pEP5289 (Dutch)
ExNg 271	3/7/2013	Remote	0.125	LS	<=0.008	S	<=0.03	S	**>=16**	**TRNG**	<=64	<=0.5	S	S	C	Aus2	12042	9716	NV5	539	13 (L421P)	23	1431	1A	19	951	953 (ISC)	6	2 (American)	Neg	17	Pos	pEp5050 (American)
ExNg 272	4/14/2013	Metropolitan	0.25	LS	<=0.008	S	<=0.03	S	<=8	Not TRNG	<=64	<=0.5	S	S	B	Aus1	7363	10117	NV7	10	13 (L421P)	166	1442	1A	230	402	808 (A39T)	6	Neg	Neg	17	Neg	Nge
ExNg 277	6/24/2013	Remote	0.125	LS	<=0.008	S	<=0.03	S	<=8	Not TRNG	<=64	<=0.5	S	S	B	Aus1	12044	10118	NV6	430	13 (L421P)	166	1443 (G120N, A121V)	1B	230	402	808 (A39T)	6	Neg	Neg	17	Pos	Nge
ExNg 278	6/24/2013	Remote	0.125	LS	<=0.008	S	<=0.03	S	<=8	Not TRNG	<=64	<=0.5	S	S	A	Aus1	12046	8063	NV17	10	13 (L421P)	166	704	1A	230	952	808 (A39T)	6	Neg	Neg	17	Pos	pEP5233
ExNg 281	6/29/2013	Metropolitan	**2**	**CMRP**	**0.03**	**DS**	**>=4**	**HLR**	<=8	Not TRNG	<=64	<=0.5	S	S		Int2	1901	4822	90	437	18 (L421P)	266 (XXXIV Mosaic)	1438 (G120K, A121N)	1B	14 (S91F, D95G)	104 (S87R)	13	3 (−35A)	Neg	Neg	18 (R251H)	Pos	Neg
ExNg 282	7/4/2013	Remote	0.25	LS	<=0.008	S	<=0.03	S	<=8	Not TRNG	<=64	<=0.5	S	S	A	Aus1	12045	7126	NV17	10	13 (L421P)	166	704	1A	230	953	811 (A39T)	6	Neg	Neg	612	Pos	Neg
ExNg 283	7/7/2013	Remote	**>2**	**PPNG**	<=0.008	S	**>=4**	**HLR**	<=8	Not TRNG	<=64	<=0.5	S	S		Int1	1890	8842	NV1	234	13 (L421P)	515	1439	1A	234 (S91F, D95A)	405 (S87N)	193 (A39T)	6	Neg	3 (blaTEM-1)	17	Neg	Neg
ExNg 285	7/17/2013	Remote	0.25	LS	<=0.008	S	<=0.03	S	<=8	Not TRNG	<=64	<=0.5	S	S	A	Aus1	12045	758	NV16	10	13 (L421P)	166	536	1A	230	953	810 (A39T)	6	Neg	Neg	612	Pos	Neg
ExNg 287	7/31/2013	Metropolitan	**2**	**CMRP**	<=0.008	S	**>=4**	**HLR**	**>=16**	**TRNG**	<=64	<=0.5	S	S		Int1	11249	10109	NV3	234	8	228	1435 (G120N, A121V)	1B	233 (S91F, D95G)	950 (S87N)	952 (ISC)	6	2 (American)	3 (blaTEM-1)	20	Pos	pEp5050 (American)
ExNg 288	7/31/2013	Metropolitan	0.5	LS	<=0.008	S	**1-2**	**R**	**>=16**	**TRNG**	<=64	<=0.5	S	S	C	Aus2	12042	9716	NV5	539	13 (L421P)	23	1431	1A	19	951	953 (ISC)	6	2 (American)	Neg	17	Pos	pEp5050 (American)
ExNg 289	7/31/2013	Remote	0.25	LS	<=0.008	S	<=0.03	S	<=8	Not TRNG	<=64	<=0.5	S	S	B	Aus1	7363	7206	NV10	10	8	20	1430 (G120N, A121V)	1B	230	947	808 (A39T)	6	Neg	Neg	20	Pos	pEP5233
ExNg 290	1/23/2011	Remote	0.125	LS	<=0.008	S	<=0.03	S	<=8	Not TRNG	<=64	<=0.5	S	S	A	Aus1	12046	8022	NV13	10	13 (L421P)	166	1434 (G120N, A121V)	1B	230	952	808 (A39T)	6	Neg	Neg	17	Pos	pEP5233
ExNg 294	8/7/2013	Remote	0.125	LS	<=0.008	S	<=0.03	S	**>=16**	**TRNG**	<=64	<=0.5	S	S	C	Aus2	12042	9716	NV5	539	13 (L421P)	23	1431	1A	19	951	953 (ISC)	6	2 (American)	Neg	17	Pos	pEp5050 (American)
ExNg 295	8/9/2013	Metropolitan	**1**	**PPNG**	<=0.008	S	<=0.03	S	<=8	Not TRNG	<=64	<=0.5	S	S		Int3	10317	5268	178	231	8	294	1444 (G120N, A121V)	1B	19	260	371 (ISC)	6	Neg	6 (blaTEM-1)	17	Pos	pEP5233
ExNg 296	8/9/2013	Remote	0.25	LS	<=0.008	S	<=0.03	S	**>=16**	**TRNG**	<=64	<=0.5	S	S	C	Aus2	12042	9716	NV5	539	13 (L421P)	23	1431	1A	19	951	953 (ISC)	6	2 (American)	Neg	17	Pos	pEp5050 (American)
ExNg 301	8/27/2013	Metropolitan	0.125	LS	<=0.008	S	<=0.03	S	<=8	Not TRNG	<=64	<=0.5	S	S		Int1	12041	10125	NV4	430	8	20	1433 (A121S)	1B	19	956	809	1	Neg	Neg	17	Pos	pEP5233
ExNg 302	9/2/2013	Metropolitan	0.125	LS	<=0.008	S	<=0.03	S	<=8	Not TRNG	<=64	<=0.5	S	S		Int1	8156	7268	NC3	470	13 (L421P)	1147 (LXIII Mosaic)	1445 (G120N, A121V)	1B	19	244	13	3 (−35A)	Neg	Neg	18 (R251H)	Neg	Neg
ExNg 304	10/1/2013	Metropolitan	0.5	LS	0.016	S	**>=4**	**HLR**	<=8	Not TRNG	<=64	<=0.5	S	S		Int2	1901	5533	90	387	19 (L421P)	266 (XXXIV Mosaic)	1440 (G120K, A121N)	1B	14 (S91F, D95G)	104 (S87R)	13	3 (−35A)	Neg	Neg	18 (R251H)	Pos	Neg
ExNg 305	10/17/2013	Remote	0.06	LS	<=0.008	S	<=0.03	S	<=8	Not TRNG	<=64	<=0.5	S	S	A	Aus1	12046	8022	NV13	10	13 (L421P)	166	1434 (G120N, A121V)	1B	230	952	808 (A39T)	6	Neg	Neg	17	Pos	Neg
ExNg 307	10/19/2013	Metropolitan	**1**	**PPNG**	<=0.008	S	<=0.03	S	<=8	Not TRNG	<=64	<=0.5	S	S		Int3	10317	5268	178	231	8	294	1446 (G120N, A121V)	1B	19	260	371 (ISC)	6	Neg	6 (blaTEM-1)	17	Pos	pEP5233
ExNg 309	10/25/2013	Remote	**1**	**CMRP**	0.016	S	<=0.03	S	<=8	Not TRNG	<=64	<=0.5	S	S		Int1	1579	21	139	231	13 (L421P)	285	1447 (G120K, A121D)	1B	381	248	13	3 (−35A)	Neg	Neg	17	Pos	Neg
ExNg 314	11/28/2013	Remote	0.125	LS	0.016	S	<=0.03	S	<=8	Not TRNG	<=64	<=0.5	S	S	C	Aus2	12043	9920	NV5	539	13 (L421P)	23	1431	1A	19	951	953 (ISC)	6	2 (American)	Neg	17	Pos	pEp5050 (American)
ExNg 316	3/8/2013	Metropolitan	0.125	LS	<=0.008	S	<=0.03	S	<=8	Not TRNG	<=64	<=0.5	S	S		Int1	7359	4186	231	537	607	23	1435 (G120N, A121V)	1B	806	804	193 (A39T)	14	Neg	Neg	20	Pos	Neg
ExNg 321	10/15/2015	Metropolitan		PPNG		S	**>=4**	**HLR**	**>=16**	**TRNG**	<=64	>256	**HLR**	S		Int1	12039	4850	NC4	456 (A2059G)	14 (L421P)	23	1241 (G120K, A121D)	1B	234 (S91F, D95A)	509 (S87R)	12 (G45D)	3 (−35A)	1 (Dutch)	2 (blaTEM-135)	17	Neg	pEP5289 (Dutch)
ExNg 322	8/9/2013	Metropolitan	0.5	LS	<=0.008	S	<=0.03	S	<=8	Not TRNG	<=64	<=0.5	S	S		Int1	8149	10120	85	234	8	20	1448 (A121S)	1B	19	949	194	1	Neg	Neg	17	Pos	pEP5233
