# Supplementary material for: Genomic epidemiology and population structure of Neisseria gonorrhoeae from remote highly endemic Western Australian populations
Source: BMC Genomics. 2018 Feb 27;19:165. doi: 10.1186/s12864-018-4557-5 (PMC6889462; doi:10.1186/s12864-018-4557-5)
Supplement: Supplementary file 3 — Table S3. List of the 72 international N. gonorrhoeae isolates from Ezewudo et al. [24]. (PDF 72 kb) [file 12864_2018_4557_MOESM3_ESM.pdf]

Table S3: List of the 72 international *N. gonorrhoeae* isolates from Ezewudo et al.

Isolate	Location	Date	MLST	Azi MIC	Cef MIC	Tet MIC	Structure group
CH811	Chile	1982	1583	0.25	0.008	2	Int1
GC1-182	Canada	1982	1583	0.5	0.008	4	Int4
SK708	Canada	2006	1594	1	0.016	0.5	Int1
SK1902	Canada	2006	10935	0.25	0.002	256	Int1
SK7461	Canada	2008	1901	0.5	0.032	8	Int1
SK7842	Canada	2006	10010	1	0.016	8	Int1
SK8976	Canada	2006	1594	0.06	0.004	2	Int1
SK12684	Canada	2006	31129	0.5	0.016	8	Int4
SK33414	Canada	2007	1928	0.25	0.008	4	Int1
SK14515	Canada	2005	1893	0.25	0.016	2	Int4
SK15454	Canada	2007	1585	0.06	0.004	2	Int3
SK16259	Canada	2007	1893	0.125	0.008	4	Int4
SK16942	Canada	2005	1893	0.125	0.016	2	Int4
SK17973	Canada	2006	1893	1	0.016	8	Int4
SK22871	Canada	2007	8122	0.125	0.004	4	Int1
SK23020	Canada	2006	1901	0.25	0.125	16	Int2
SK28355	Canada	2007	1893	0.25	0.016	4	Int4
SK29344	Canada	2007	10010	0.125	0.008	4	Int1
SK29471	Canada	2005	1893	0.25	0.016	2	Int4
SK32402	Canada	2007	8153	0.5	0.016	4	Int2
SK36809	Canada	2007	8126	2	0.008	8	Int4
SK39420	Canada	2008	1585	0.5	0.016	0.5	Int3
ALB0303	USA	2011	1588	0.03	0.015	16	Int1
ALB0403	USA	2011	1901	1	0.125	4	Int2
ATL0103	USA	2011	10931	0.5	0.015	0.25	Int1
ALB0102	USA	2011	1901	0.25	0.06	2	Int2
ATL0105	USA	2011	1588	0.06	0.015	0.25	Int1
ATL0108	USA	2011	1584	0.03	0.015	0.25	Int3
ATL0117	USA	2011	10932	0.125	0.015	16	Int1
ATL0121	USA	2011	1902	0.5	0.03	1	Int1
ATL0125	USA	2011	1901	0.25	0.015	1	Int2
ATL0508	USA	2011	1585	0.06	0.015	16	Int3
ATL0513	USA	2011	1893	0.25	0.03	2	Int4
MIA0202	USA	2011	1901	0.5	0.03	2	Int1
MIA0309	USA	2011	1931	0.125	0.015	16	Int3
MIA0310	USA	2011	1584	0.03	0.015	16	Int3
MIA0510	USA	2011	1901	1	0.03	2	Int2
MIA0515	USA	2011	1901	0.25	0.03	16	Int2
MIA0516	USA	2011	1901	0.5	0.06	8	Int1
NOR0306	USA	2011	1583	0.25	0.015	2	Int1
NYC0507	USA	2011	1901	0.25	0.06	2	Int2
NYC0513	USA	2011	1901	0.25	0.06	4	Int1
MUNG1	Canada	1991	10934	0.125	<0.016	0.25	Int1
MUNG3	Japan	2003	7363	0.25	0.5	2	Int1
MUNG4	Japan	1996	1590	0.5	0.25	4	Int1
MUNG5	Philippines	1992	1901	0.25	<0.016	1	Int2
MUNG6	Australia	2001	10008	0.125	<0.016	16	Int1
MUNG8	USA	2001	8127	2	<0.016	0.5	Int4
MUNG9	Sweden	2010	1901	0.5	1	2	Int2
MUNG12	Norway	2010	1901	0.5	0.25	4	Int2
MUNG14	Norway	2010	1901	0.5	0.25	4	Int2
MUNG15	Austria	2011	1901	0.25	1	2	Int2
MUNG17	Sweden	2010	1892	1	0.5	2	Int1
MUNG18	Norway	2010	10933	0.125	<0.016	2	Int1
MUNG19	Sweden	2010	1580	>256	<0.016	2	Int4
MUNG20	Sweden	2013	7363	0.25	0.5	2	Int1
MUNG21	Pakistan	2008	1902	1	0.032	2	Int1
MUNG23	Sweden	1998	1585	0.064	<0.016	0.125	Int3
MUNG25	Sweden	1998	1901	0.125	<0.016	0.5	Int2
MUNG26	Sweden	1999	1584	0.064	<0.016	0.5	Int3
FA1090			1899				Int1
FA19			1892				Int2
NCCP11945	South Korea		1901				Int2
DGI2	USA		8421				Int1
PID24	USA		8418				Int1
DGI18	USA		8418				Int1
FA6140	USA		1927				Int1
PID18	USA		1926				Int1
NG1291	USA		8422				Int1
F62			1900				Int1
PID332	USA		1594				Int1
MS11			6959				Int1

RS. 2015. Population structure of Neisseria gonorrhoeae based on whole genome data and its relationship with antibiotic resistance. PeerJ 3:e806.
